# Protective effect of anakinra on audiovestibular function in a murine model of endolymphatic hydrops

**DOI:** 10.3389/fncel.2022.1088099

**Published:** 2022-12-15

**Authors:** Na Zhang, Na Li, Siyue Wang, Wandi Xu, Jiahui Liu, Yafeng Lyu, Xiaofei Li, Yongdong Song, Ligang Kong, Yalan Liu, Jia Guo, Zhaomin Fan, Daogong Zhang, Haibo Wang

**Affiliations:** ^1^Department of Otolaryngology-Head and Neck Surgery, Shandong Provincial ENT Hospital, Shandong University, Jinan, Shandong, China; ^2^Shandong Provincial Vertigo and Dizziness Medical Center, Jinan, Shandong, China; ^3^Laboratory of Vertigo Disease, Shandong Second Provincial General Hospital, Shandong Institute of Otorhinolaryngology, Jinan, Shandong, China; ^4^Center of Clinical Laboratory, Shandong Second Provincial General Hospital, Jinan, Shandong, China

**Keywords:** anakinra, IL-1β, endolymphatic hydrops, Ménière’s disease, demyelination

## Abstract

**Introduction:**

Ménière’s disease (MD), a common disease in the inner ear, is characterized by an increase in endolymph in the cochlear duct and vestibular labyrinth. The pathophysiology of the condition appears to be the immune response. Studies have shown that basal levels of the IL-1β increased in some MD patients.

**Methods:**

Here, we used a murine model of endolymphatic hydrops (EH) to study the effect of anakinra on auditory and vestibular function. Mice were intraperitoneal injected with anakinra or saline before LPS by postauricular injection. Weight and disease severity were measured, histologic changes in auditory were assessed, and inflammation state was evaluated.

**Results:**

We found that anakinra therapy reduced LPS-induced EH, alleviated LPS-induced hearing loss and vestibular dysfunction, and inhibited the expression of the inflammatory cytokines and macrophage infiltration in the cochlea of mice. We further demonstrated that anakinra ameliorated the disorganization and degeneration of myelin sheath, and reduced the neuron damage in cochlea of EH mice.

**Discussion:**

Consequently, anakinra contributes to a promising therapeutic approach to MD, by restricting EH, alleviating auditory and vestibular function, inhibiting inflammation of the inner ear and protecting the cochlear nerve. Further investigations are needed to assess the potential therapeutic benefits of anakinra in patients with MD.

## 1 Introduction

Ménière’s disease (MD), a complex peripheral vestibular illness, is characterized by episodic vertigo, fluctuating low-to-medium frequencies of sensorineural hearing loss, tinnitus, and/or aural fullness (Sajjadi and Paparella, 2008). Population-based studies report that MD affects about 3.5–513 people per 100,000 annually (Alexander and Harris, 2010). Endolymphatic hydrops (EH) underlies its classic pathological characteristics (Hallpike and Cairns, 1938; Okuno and Sando, 1987). Numerous underlying factors have been postulated to be involved in MD, including anatomical variations in the temporal bone, genetic heterogeneity, allergies, immune dysfunction, and cellular and molecular mechanisms (Derebery and Berliner, 2010; Gazquez et al., 2011; Greco et al., 2012; Requena et al., 2014; Nakashima et al., 2016; Na Zhang et al., 2022). Because the mechanisms underlying MD remain poorly understood, the clinical treatment strategy of MD is mainly to relieve the symptoms. Patients after treatment still suffer from recurrent vertigo and progressive hearing loss, and need hospital treatment frequently, which seriously affects the psychological status, quality of life and economic burden of patients (Tyrrell et al., 2016). Therefore, it is of great practical significance to study the pathogenesis and open up a more accurate treatment direction of MD.

Tissue-resident macrophages play critical roles in inflammation regulation for specific organ by releasing interferons, inflammatory cytokines, and chemokines. Studies have showed that there is a large population of macrophages in the inner ear, can be recruited from blood-borne monocytes to hair cells following damage induced by noise, ototoxic drugs, and aging (Liu et al., 2018), which may be important to initiate local inflammatory response in the cochlear and vestibular system (Zhang et al., 2013; O’Malley et al., 2016; Nakanishi et al., 2017).

Recent studies have shown that immunology is an important pathogenic factor of MD, although the molecular mechanisms remain poorly understood. It is estimated that a third of patients with MD may have immune dysfunction (Greco et al., 2012). Our previous study suggested that MD is in a state of inflammation manifested in elevated levels of Th2-relative cytokines such as interleukin (IL)-4, IL-5, IL-10, and IL-13 in serum, vestibular end organ (VEO) and endolymphatic sac (ES) (Zhang et al., 2022). IL-1β, exerts strong proinflammation, overproduction by macrophages causes autoinflammatory diseases (Gabay et al., 2010; Canna et al., 2014; Jesus and Goldbach-Mansky, 2014). A study reported that compared to healthy controls, the levels of pro-inflammatory cytokines such as IL-1β, IL1-receptor antagonist (IL-1RA), tumor necrosis factor alpha (TNFα), and IL-6 released by peripheral blood mononuclear cells are elevated in 24 of 113 patients (20%) with MD (Frejo et al., 2018). Additionally, polymorphisms in IL-1 are associated with MD (Furuta et al., 2011; Li et al., 2017; Kouhi et al., 2021). These studies suggest that IL-1β may play an important role in MD pathogenesis and IL-1β-targeted therapy may be a potential treatment for MD.

IL-1RA blocks the biological cascades and inhibits the biological effects of IL-1, acting as a natural decoy for IL-1β (Dinarello, 2018). Anakinra, a recombinant form of IL-1RA, exerts anti-inflammatory and immunomodulatory roles. In 2001, it was approved by the US Food and Drug Administration for the treatment of rheumatoid arthritis (Garces, 2001), and has shown significant therapeutic effects associated with an unparalleled record of safety on a broad spectrum autoimmune and inflammation diseases such as steroid-refractory immune effector cell-associated neurotoxicity syndrome (Wehrli et al., 2022), Coronavirus disease 2019 (Kooistra et al., 2020), and gout flares (Saag et al., 2021). Moreover, anakinra has been demonstrated to reverse or improve hearing loss and inflammatory of an atypical cryopyrin-associated periodic syndromes (Nakanishi et al., 2017). However, previous studies have neither revealed its role in MD development nor its regulatory mechanisms. Although MD is considered closely related to inflammation, whether anakinra have therapeutic effect on MD remains unexplored.

Animal models are an important tool for exploring the pathogenesis and treatment of diseases. At present, models of chronic EH have been established by surgical obstruction of the ES or systemic drugs such as aldosterone, vasopressin, or lipopolysaccharide (LPS) for cochlear and vestibular research (Seo and Brown, 2020). For example, LPS induces moderate to severe hearing loss and EH (Takumida et al., 2008; Brown et al., 2018), and contributes to a better understanding of the MD pathogenesis.

In this study, we investigated whether blockade of IL-1β signaling by anakinra could inhibit EH and audiovestibular symptoms in LPS-induced EH murine model. We further focused on the alterations of inflammatory response and cochlear nerve. Taken together, this points to a potential and promising therapeutic strategy for MD.

## 2 Materials and methods

### 2.1 Mice and LPS injection

Wild type C57BL/6 mice (purchased from the Animal Center of Shandong university) were housed in a temperature-controlled (20–22°C) room that allows for a 12/12 h light/dark cycle. Sex-matched mice had access to food and drinking water, weighed 17–25 g, were 8–10 weeks old. All study protocols were approved by the Animal Care Committee of Shandong university and conformed to the National institutes of Health Guide for the Care and Use of Laboratory Animals.

C57BL/6 mice were subjected to LPS-induced EH. Briefly, mice were challenged with LPS (2 mg/kg, Sigma) solvent in saline by bilateral postauricular injection (p.a.) (Li et al., 2013) once a day for 3 days, while control groups were p.a. injected with equivalent 0.9% normal saline (NS).

### 2.2 Anakinra injection

To evaluate the roles for anakinra in LPS-induced EH, mice were intraperitoneal injected (i.p.) with anakinra (10 mg/kg, Med Chem Express, Monmouth Junction, NJ, USA) or equivalent NS 30 min before LPS challenge. In all cases, hearing and vestibular function were tested on day 5 after first dose of LPS injection. After then, those mice were anesthetized by intraperitoneal injection with a mixture of xylazine (10 mg/kg) and ketamine (100 mg/kg) and inner ears were subjected to protein or RNA expression and EH analysis.

### 2.3 Frozen sections

The mouse cochleae were collected, fixed with 4% paraformaldehyde overnight at 4°C, continuously dehydrated in a sucrose phosphate-buffered saline mixture with sucrose concentrations increasing at 15, 20, and 30%, and ultimately encapsulated in an OCT compound (Tissue−Tek, Sakura Finetek, Torrance, USA). The embedded cochlea was sliced (5 μm thick) using a cryostat (Leica CM 1850, Nussloch, Germany) and the slice was stored at −80°C.

### 2.4 Histological and histomorphometric analyses

For hematoxylin-eosin (HE) staining, the cochlear sections were stained with hematoxylin (H8070, Solarbio life sciences, Beijing, China) for 3 min followed 2 min of staining with eosin (C0109, Beyotime, Shanghai, China) at room temperature.

For the immunofluorescence, the cochlear sections were permeabilized in 0.5% Triton X-100 for 30 min, and blocked with 10% donkey serum for 60 min at room temperature. The sections were incubated with the appropriate primary antibodies at 4°C overnight: anti-CD68 antibody (1:200; 97778, CST, Boston, USA), anti-Tuj1 (1:800; 5568, CST, Boston, USA), anti- Myelin Basic Protein (MBP, 1:200; 78896, CST, Boston, USA), and anti-Neurofilament 200 antibody (NF-200, 1:20; 2836, CST, Boston, USA). At last, the sections were incubated with the relevant fluorescently labeled secondary antibodies (1:1,000; Invitrogen, Carlsbad, CA, USA) and DAPI (1:1,000; D9542; Sigma-Aldrich, St. Louis, MO, USA) for 60 min in dark at room temperature. The sections were photographed under a laser scanning confocal microscope (Leica SP8; Leica, Wetzlar, Germany).

### 2.5 Rotarod test

The mice were placed on an electric rotating rod (ZH–600 B, Huaibei Zhenghua Biological Instruments Co., Ltd.). Gradually increased the speed to 30 rpm for 2 min. Each mouse was trained 2 times per day for 3 days and tested twice. Whereafter, the average time to fall off the rotarod in 2 trials was calculated for analysis.

### 2.6 Swim test

General vestibular function could be scored using swimming tests (Clopath et al., 2014). Mice were placed in a standard pool with a water level height of approximately 15 cm and a water temperature of around 25°C. Mice were scored for swimming posture, with mice swimming in the water (score 0), swimming irregularly (score 1), floating stationarily (score 2), or rolling underwater (score 3).

### 2.7 Vestibular evoked myogenic potentials (VEMPs)

Click-evoked VEMPs was recorded at the same time as electromyography potential after anesthesia. A custom-made holder previously reported (Sheykholeslami et al., 2009) was used to fix the mice with a suspension wire behind the anterior teeth, so that the mice hyperextended their necks and raised their heads with free legs, holding them in a prone position. For the test, a needle electrode was inserted into the cervical extensor muscle and a reference electrode was placed in the midline of the cervico-occipital region, with the ground electrode placed on the back. Each animal was tested for VEMPs with a stimulation intensity of 100 dB nHL. The response threshold is the minimum threshold at which the waveform occurred. Its repeatability was verified by running it continuously (>3 times). Eventually, the latencies and amplitudes of the negative and positive peaks were recorded.

### 2.8 Vestibular ocular reflexes (VOR)

VOR test was conducted as reported previously (Yang et al., 2019). Briefly, mice were placed with a non-invasive animal-immobility setup. Horizontal eye position signals were recorded using a binocular VOG-based VFT system provided by Prof. Fangyi Chen’s team (Southern University of Science and Technology). An infrared camera equipped with a zoom lens (MI^®^, China) was placed on translation stages at 45° angles to the anteroposterior axis of the mouse. Illumination of the video recording was achieved by two near-infrared light-emitting diode lamps (940 nm, Chuntaxin^®^, China) that were connected to the camera. The eye tracker tracked the region of interest (ROI) at a speed of 60 frames/s. ROI, which contains the pupil, in each frame was automatically selected following a template-matching method. Then, an ellipse fit was used to determine the pupil center, and to extract the horizontal component derived from the eye movements. The obtained translation distances were converted to eye rotation angel for calibration. To measure the VOR responses, horizontal rotations were delivered at 20°/s (0.2, 0.5, 0.8, 1.0, 1.6, and 3.2 Hz). Exported eye-position data underwent Fourier transformation using the MATLAB 2016b software to yield amplitude data for eye movement. The VOR gain were calculated as the amplitude ratio between response and stimulus.

### 2.9 Auditory function evaluation

The auditory brainstem response (ABR) were measured as previously described (Zhang et al., 2020). The sound level started at a 90-dB sound pressure level (SPL) and then decreased by 5-dB to the acoustic threshold. The ABR threshold was determined at each frequency, which refers to the minimal SPL resulting in a reliable ABR recording with one or more distinguishable waves clearly identified by visual inspection. Repeated the process for low SPLs near the threshold to ensure the waveform consistency. Mice were housed in a sound isolation chamber and stimulated at sound frequencies of 4, 8, 12, 16, 24, and 32 kHz, each frequency to be repeated 1,024 times, and ABR responses were measured using the TDT System 3 (Tucker-Davis Technologies, Alachua, FL, USA). Briefly, a total of 15 mice (*n* = 5 per group) were anesthetized by intraperitoneal injection with a mixture of xylazine (10 mg/kg) and ketamine (100 mg/kg). The speaker was positioned 10 cm away from each mouse. Needle electrodes are inserted into the subcutaneous tissue of the mouse. The recording electrode is inserted into the cranial vault, the reference electrode is inserted in the subauricular mastoid region of the ipsilateral ear, and the grounding electrode is inserted on the back. Starting from a SPL of 90 dB and decreasing by 5 dB to the hearing threshold. The ABR threshold determined at each frequency was the minimum SPL that produced a reliable ABR recording where the eye could clearly identify the waveform. This process was repeated for lower SPLs close to the threshold to ensure consistency of the waveform.

### 2.10 Extraction of mRNA and quantitative real-time PCR (qRT-PCR)

Total RNA of the mouse cochlea was obtained using the RNA extraction kit (RNeasy Mini QIAcube Kit, QIAGEN, Hilden, Germany). The relative expression levels of RNA were calculated by reverse transcription and assayed using qRT-PCR (Eppendorf AG 22331 PCR, Hamburg, Germany). The qRT-PCR reaction mixture comprised 2 × SYBR Green Premix EX Taq (RR42LR, Takara Biotechnology, Shiga, Japan), cDNA template, forward and reverse primer, and deionized water. The qRT-PCR parameters involved an initial denaturation step at 95°C for 3 min, then denaturing at 95°C for 50 s, 60°C with annealing for 45 s, and a further extension at 72°C for 50 s across 40 cycles. The specificity of each PCR reaction was identified by the melting curve analysis. The primers used in this experiment were listed in Table 1. *Actin* was used to standard the target mRNA expression. The fold change for each gene was measured with the comparative Ct method (Zhang et al., 2020).

**TABLE 1:** Primer sequences for quantitative RT-PCR.

Gene	Forward primers	Reverse primers
Actin	GTCCCTCACCCTCCCAAAAG	GCTGCCTCAACACCTCAACCC
Ilb	GAAATGCCACCTTTTGACAGTG	TGGATGCTCTCATCAGGACAG
Ila	TTGGTTAAATGACCTGCAACA	GAGCGCTCACGAACAGTTG
Il6	GGAGCCCACCAAGAACGATAGTCAA	GTCACCAGCATCAGTCCCAAGAA
Tnf	CAGGCGGTGCCTATGTCTC	CGATCACCCCGAAGTTCAGTAG
Ifnb	CAGCTCCAAGAAAGGACGAAC	GGCAGTGTAACTCTTCTGCAT
Il4	CCAAACGTCCTCACAGCAAC	AGGCATCGAAAAGCCCGAA
Il5	AATCAAACTGTCCGTGGGGG	CTCTCCTCGCCACACTTCTC
Il8	GGCTTTGCGTTGATTCTGGGAACT	AGCGGTGTCCTGATTATCGTCCT
Mmp3	TTTGATGCAGTCAGCACCCTCCG	TCGTGCCCTCGTATAGCCCAGAA
Mmp13	TCACCTGATTCTTGCGTGCTATG	CTTTATCTGTGCTCATCTGTGGC

### 2.11 Enzyme linked immunosorbent assay (ELISA)

Cytokine concentrations were determined using mouse IL-1β ELISA kit (ab197742; Abcam, Cambridge, MA, USA) depending on the manufacturer’s instructions. The signal was examined at 450 nm using a microplate reader (Bio-RAD).

### 2.12 Quantitative assessment of changes in endolymphatic space in cochlea

For quantitative assessment of the changes of EH in each cochlea, the length of the stretched Reissner’s membrane (L) and the ideal length of the Reissner’s membrane (L*) were measured as stated in the literature before (Katagiri et al., 2014). The increase ratio of the length change of the Reissner’s membrane (IR-L = L/L* × 100%) was calculated to evaluate the level of EH in upper and lower turns. Image J software was used for the above measurements.

### 2.13 Quantitative evaluation of immunofluorescence staining

All quantification in the study was performed in a blinded fashion. Three fields that covered a similar area were obtained for the mid-modiolar cochlear sections. Images were analyzed using ImageJ v1.51. The eight-bit blue channel image was corrected for background illumination using the eight-bit blue channel of a bright-field image (Zhang et al., 2022). A threshold was then applied to the image to measure the total expression identified through specific immunostaining. The integrated optical density (IOD)/pixel of the fields was used to indicate the CD68 load and the expression of MBP and FN-200.

### 2.14 Statistical analysis

SPSS 13.0 software (SPSS Inc., USA) was applied for statistical analysis. All data are shown as the mean ± standard deviation (SD). One-way ANOVA followed by a Dunnett multiple comparisons test was used to compare more than 2 groups. *P*-values < 0.05 were regarded as statistically significant differences.

## 3 Results

### 3.1 Anakinra reduced the severity of LPS-induced EH in mice

**FIGURE 1:**
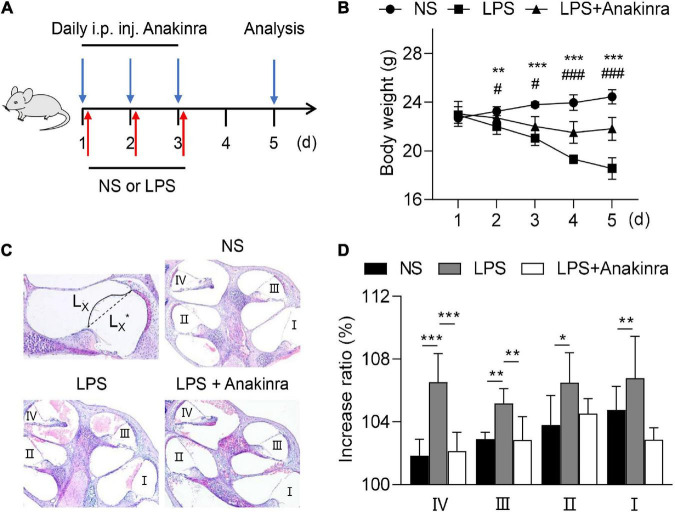
Anakinra reduced the severity of LPS-induced endolymphatic hydrops in mice. **(A)** Mice were untreated or pretreated treated with Anakinra (10 mg/kg) and challenged with LPS (10 mg/kg, i.p.) or equivalent saline for 3 consecutive days, then sacrificed at 5 days. **(B)** Changes of body weight in all groups (*n* = 5, *NS group compared to LPS group, ^#^LPS group compared to LPS + AN group). **(C)** Representative images of mid-modiolar cochlear sections in all groups. **(D)** Measurements of the increase rations of the length of the Reissner’s membrane in cochlear half-turns I–IV (*n* = 5). Data are presented as mean ± SD. *or ^#^*p* < 0.05, **< 0.01 ***or ^###^*p* < 0.001. NS, 0.9% normal saline; i.p. inj., intraperitoneal injected.

Based on previous studies, LPS was injected subcutaneous behind the ear to induce EH model (Brown et al., 2018) to determine whether anakinra effects the clinically relevant MD process. Anakinra (i.p.) and/or LPS (p.a.) was injected to age- and sex-matched C57BL/6 mice for 3 consecutive days, and then we assessed the features of relevant EH and audiovestibular symptoms thus induced (Figure 1A). Ranging from 1 to 5 days, the body weight of control mice increased slowly, while the body weight in the LPS group decreased, which was significantly different from that in the control group from the second day (Figure 1B). Compared with LPS group, anakinra treatment significantly decreased the weight loss induced by LPS (Figure 1B). Here, we quantified and evaluated the EH level. As shown in Figures 1C, D and the LPS-mice showed enlargement of scala media and more severe EH in all cochlear turns than control mice. Next, we analyzed whether anakinra reduced the outcomes of LPS-induced EH. Indeed, we found that LPS mice treated with anakinra result in alleviating of EH in cochlear upper-turns, not in lower-turns (Figures 1C, D). These data showed that anakinra decreased the severity of hydrops in LPS-induced EH mouse model.

### 3.2 Anakinra alleviated LPS-induced vestibular dysfunction in mice

**FIGURE 2:**
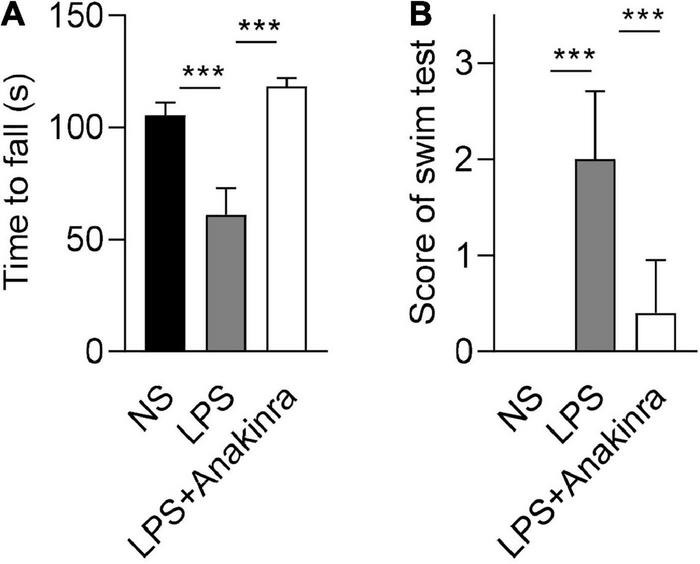
Anakinra alleviated LPS-induced vestibular dysfunction in mice. **(A)** Quantification of rotarod test (*n* = 5). **(B)** Quantification of swim tests (*n* = 5). Data are presented as mean ± SD. ***p < 0.001. NS, 0.9% normal saline.

**FIGURE 3:**
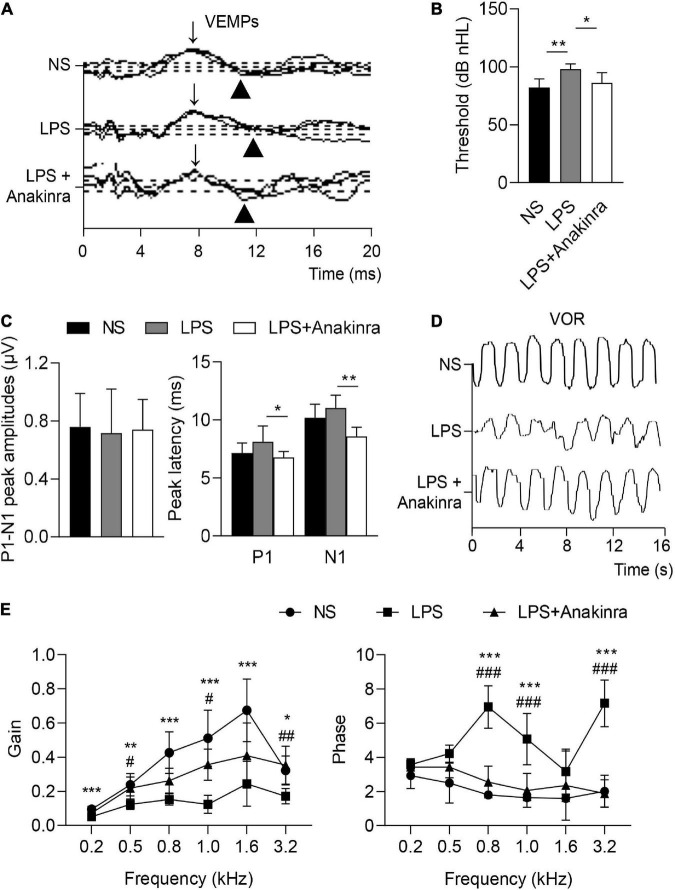
Anakinra alleviated LPS-induced vestibular dysfunction in mice. **(A)** Representative click-evoked VEMPs waves, P1(**↓**) and N1(▲). **(B)** Changes in VEMPs threshold (*n* = 5). **(C)** Changes in P1-N1 peak amplitudes, and the peak latencies of P1 and N1 at 100 dB nHL (*n* = 5). **(D)** Representative horizontal VOR waves of all groups. **(E)** The VOR gains and phases are plotted for all groups at a peak velocity (20°/s) and frequencies range from 0.2 to 3.2 kHz (*n* = 5, *NS group compared to LPS group, ^#^LPS group compared to LPS + Anakinra group). Data are presented as mean ± SD. *or ^#^*p* < 0.05, **or ^##^< 0.01 ***or ^###^*p* < 0.001. NS, 0.9% normal saline; VEMPs, vestibular-evoked myogenic potentials; P1, positive peak; N1, negative peak.

Vertigo caused by vestibular dysfunction is one of main clinical symptoms of MD, we next performed experiments to determine whether anakinra alleviated the vestibular symptoms of LPS-induced EH murine model. To evaluate the vestibular function, we performed behavioral tests and electrophysiological examination for mice (Nist-Lund et al., 2019). The mice were subjected to rotarod tests and the time remained on the rotating rod with increasing acceleration were recorded. After training, LPS-challenged mice performed shorter latency time to fall from accelerating rotarod than the control mice, while LPS mice treated with anakinra increased the residence time on the rotarod (Figure 2A and Supplementary Video 1). For swim test, the control group displayed normal swimming (score 0), while the scores were significantly raised for LPS-treated mice, and reversed after anakinra treatment (Figure 2B). The positive peak (P1, **↓**) and negative peak (N1, ▲) latency in VEMPs in response to clicks presented at 100 dB nHL were performed and shown in Figure 3A. After administration of LPS, the mice showed increased threshold compared to those control mice, but no differences in the threshold or amplitudes between P1 and N1 or latency of P1 and N1 (Figures 3B, C). Indeed, anakinra treatment decreased the threshold and latency of P1 and N1 (Figures 3B, C). We further examined the function of horizontal semicircular canal using horizontal VOR test delivered at 20°/s (0.2, 0.5, 0.8, 1.0, 1.6, and 3.2 Hz), and the VOR gain and phase were calculated (Figure 3D). The LPS group showed a significant decrease of VOR gain from 0.2 to 3.2 Hz, and the increased phase was identified at 0.8, 1.0, and 3.2 Hz compared to NS group (Figure 3E). In contrast, LPS mice treated with anakinra resulted in reversing the gain decreases and phase increases (Figure 3E). Together, these data showed that anakinra alleviated damage to vestibular function in LPS-induced EH mouse model.

### 3.3 Anakinra ameliorated LPS-induced hearing loss in mice

**FIGURE 4:**
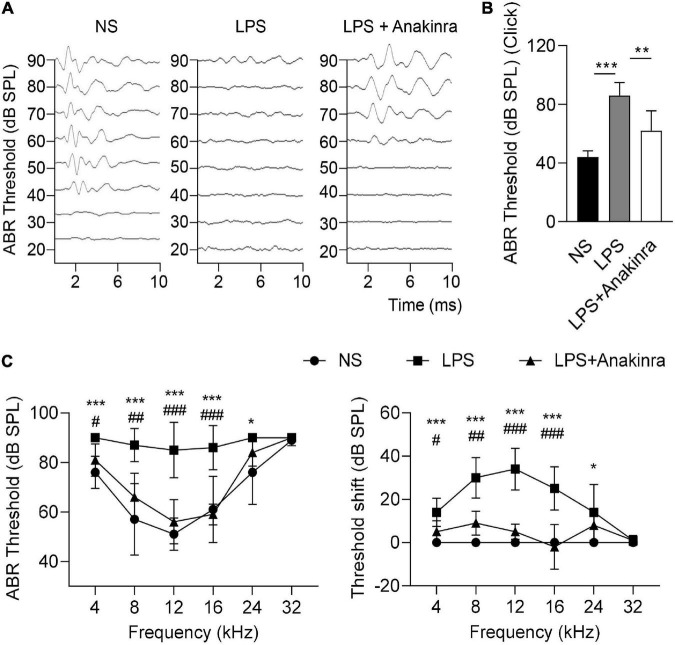
Anakinra ameliorated LPS-induced hearing loss in mice. **(A)** Representative serial ABR wave recordings in response to click sounds (*n* = 5). **(B)** ABR thresholds in response to click sounds (*n* = 5). **(C)** ABR thresholds and threshold shift in response to pure tone bursts across all frequencies tested (4, 8, 12, 16, 24, and 32 kHz) (*n* = 5). Data are presented as mean ± SD. *or ^#^*p* < 0.05, **or ^##^< 0.01 ***or ^###^*p* < 0.001. NS, 0.9% normal saline; ABR, auditory brainstem response.

To evaluate the auditory function, we measured click-evoked and tone burst-evoked (8–32 kHz) ABR. The results showed that click-induced ABR threshold were considerably higher in LPS mice compared to saline group, these effects were significantly reversed by anakinra therapy (Figures 4A, B). For tone burst-induced ABR, LPS induced the threshold and threshold shift increases in the frequency range from 8 to 24 kHz, not in 32 kHz (Figure 4C), which consistent with the clinical manifestation of low-to-medium frequencies of sensorineural hearing loss in patients with MD. Indeed, we found that mice treated with anakinra reversed threshold and threshold shift increases in the frequency range from 8 to 16 kHz caused by LPS (Figure 4C). Altogether, these data showed that anakinra ameliorated damage to auditory function especially in low-to-medium frequencies in LPS-induced EH mouse model.

### 3.4 Anakinra inhibited LPS-induced inflammation and macrophage infiltration

**FIGURE 5:**
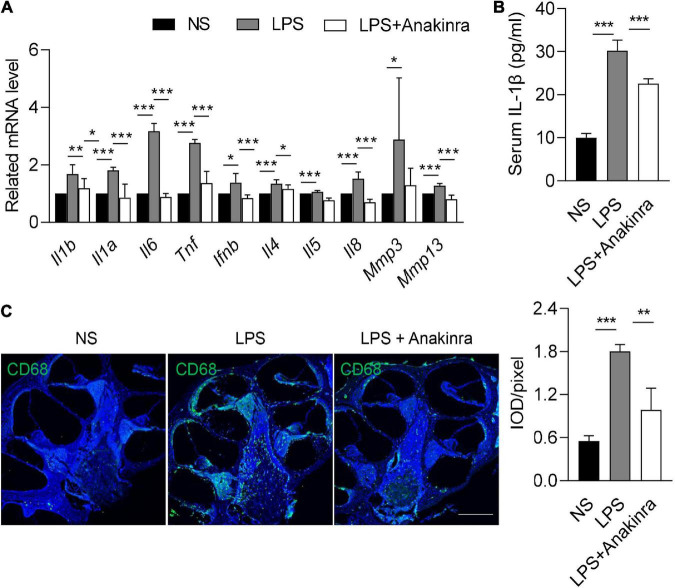
Anakinra inhibited LPS-induced inflammation and macrophage infiltration. **(A)** Related mRNA expression level in cochlea (*n* = 5). **(B)** ELISA for serum IL-1β levels (*n* = 5). **(C)** Immunofluorescence staining and quantification for CD68 (green) and DAPI (blue) in mid-modiolar cochlear sections. Analysis of images is shown at right, quantifying the load of CD68 per pixel (*n* = 3), scale bar = 500 μm. Data are presented as mean ± SD. *p < 0.05, **< 0.01 ***p < 0.001. NS, 0.9% normal saline.

As per our previous report (Zhang et al., 2022), VEO and ES of patients with MD were associated with elevated expression of inflammatory cytokines. Microarray analysis demonstrated the increased expression levels of inflammatory cytokines in the cochlea of mice treated with LPS (Lee et al., 2022). Consistent with these findings, mRNA expressions of inflammatory cytokines *Il1b*, *Il1a*, *Il6*, *Tnf*, *Ifnb*, *Il4*, *Il5*, and *Il8* were elevated in the cochlea of LPS-treated mice (Figure 5A). Elevated matrix metalloproteinase-3 (MMP3) level affects hearing function (Nasution and Haryuna, 2019). Real-time PCR also revealed that the mRNA levels of *Mmp3* and *Mmp13* were upregulated in the cochlea of LPS-treated mice relative to control mice (Figure 5A). Intervention with anakinra decreased the mRNA expression levels of any of the above genes in LPS-treated mice (Figure 5A). In addition, our results showed that the serum IL-1β in LPS-treated mice was significantly increased, and effectively reduced when intervention with anakinra (Figure 5B).

Macrophage is the main group and considered to play an important role in the microenvironment of the cochlea (Kampfe Nordstrom et al., 2018). We next performed immunofluorescence to determine if anakinra similarly reduced macrophages numbers in the cochlea. Compared with control mice, LPS-induced EH was associated with elevated numbers of macrophages in the cochlea (Figure 5C). Indeed, LPS mice treated with anakinra attenuated the infiltration of macrophages (Figure 5C). Together, these data suggested that anakinra inhibited inflammation response and macrophage infiltration in LPS-induced EH mouse model.

### 3.5 Anakinra protected LPS-induced neurons and myelin sheath damage

**FIGURE 6:**
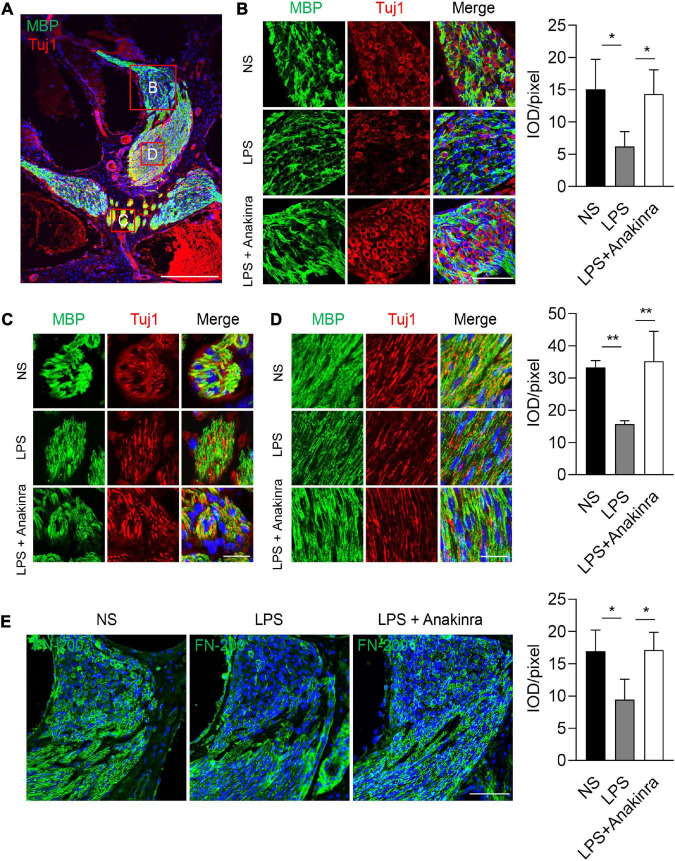
Anakinra protected LPS-induced neurons and myelin sheath damage. **(A)** Low-power view of mid-modiolar cochlear sections from LPS group illustrating auditory nerve fibers in different regions stained positively for MBP (green) and Tuj1 (red). Nuclei were counterstained with DAPI (blue), scale bar = 500μm. **(B)** Confocal images of SGNs for MBP (green), Tuj1 (red), and DAPI (blue) within the medial portion of Rosenthal’s canal in the upper turns of mice. Analysis of images is shown at right, quantifying the expression of MBP per pixel (*n* = 3), scale bar = 50 μm. **(C, D)** MBP^+^ nerve fibers are illustrated in central portions of the auditory nerve in the ears. Analysis of images is shown at right, quantifying the expression of MBP per pixel (*n* = 3), scale bar = 12.5 μm. **(E)** Immunofluorescence staining for FN-200 (green) and DAPI (bule) in cochlear axis. Analysis of images is shown at right, quantifying the expression of FN-200 per pixel (*n* = 3), scale bar = 50 μm. Tuj1, class III β -tubulin. Data are presented as mean ± SD. *p < 0.05, **< 0.01. NS, 0.9% normal saline.

MBP is one of the major myelin protein produced by oligodendrocytes and Schwann cells in the nervous system, and plays a critical role in maintaining the compact structure of the myelin sheath (Simons and Trotter, 2007). Class III β-tubulin (Tuj1) stains the microtubule components of type I spiral ganglion neurons (SGNs) (Liu et al., 2021b). To evaluate the effects of anakinra on cochlear nerve, we performed immunofluorescence staining to analyze myelination of SGN fibers and nerve fiber architecture using anti-MBP and anti-NF-200-antibodies, respectively. Focusing on the center of SGNs, we found that MBP surrounded SGNs and their processed (Figure 6A). In control mice, high-resolution confocal images revealed strong immunoreactivity of MBP was seen in the central myelinated processes of the cochlear nerve, and most SGNs were intact and enclosed by a MBP^+^ myelin sheath (Figures 6B–D). In the LPS-treated mice, MBP was expressed in the same locations as in control mice. However, abnormalities in the staining pattern and weaker immunostaining reaction for MBP were seen in the SGNs of LPS mice compared to that in NS control mice. Numerous disruptions were present in the MBP^+^ myelin sheath surrounding LPS SGNs in cochlear upper turn suggesting an excess of demyelinated axons and decreased myelin integrity in LPS-treated SGNs (Figure 6B). The MBP^+^ myelin sheaths, Tuj1^+^ terminations and axons in many neurons were discontinuous and in some cases appeared to be missing altogether (Figures 6C, D). In addition, LPS mice treated with anakinra upregulated the expression of MPB and reduced the loss of myelin sheath and SGNs. NF-200 is a neuronal structural protein (Chen et al., 2017). A marked decrease in immunostaining intensity for NF-200 was seen in SGNs of LPS group compared to NS group, while anakinra reversed the loss of NF-200 signal (Figure 6E). Together, these results demonstrated that anakinra protected structural damage of cochlear nerve in EH mouse cochlea.

## 4 Discussion

IL-1β is a strong pro-inflammatory cytokine which is mainly produced by activated macrophages and is widely regarded as a hallmark of inflammatory gene cascades (Weber et al., 2010). Consistent with high basal levels of IL-1β released by peripheral blood mononuclear cells in MD (Frejo et al., 2018), our study found that LPS induced abnormal increase of IL-1β and macrophage infiltration in EH murine model, accompanied by elevated serum IL-1β. Many immune-related diseases are accompanied by immune imbalance and increased pro-inflammatory cytokines (Smolen et al., 2005; Tian et al., 2020), which is also found in our MD-relevant murine model. The findings further suggest that IL-1β signal plays an important role in the pathogenesis of MD.

The abnormal IL-1β in the inner ear may be secreted by macrophage. Macrophage is the main group and is considered to play an important role in the innate and adaptive defense and tolerance in the inner ear (Kampfe Nordstrom et al., 2018; Liu et al., 2021a). Resident macrophages in inner ear proliferate slowly, and renewal is primarily by the infiltration and differentiation of circulating bone-marrow-derived cells (Shi, 2010), whether IL-1β in the inner ear is secreted by resident macrophages or by macrophages derived from bone marrow cells is remain unknown. Studies have shown that macrophages effect blood/labyrinth barrier integrity, and loss of macrophages is associated with tissue edema (Zhang et al., 2012). More importantly, LPS and ototoxic drugs activate macrophage and initiate local inflammatory response in the inner ear (Zhang et al., 2013, 2020; Nakanishi et al., 2017). Moreover, whether the local immune disorder leads to the systemic immune imbalance, or the local abnormality caused by the systemic imbalance remains to be elucidated, which need further study.

IL-1 receptor (IL-1R) promotes or inhibits inflammation and immune response, notably innate immune reactions, and the downstream signal transduction regulates the expression of various proinflammatory cytokines, such as IL-1, IL-6, IL-8, and TNF-a (Narayanan and Park, 2015). IL-1RA binds to IL-1R with a high affinity and competitively interferes with the binding of IL-1 to IL-1R as an inhibitory protein for IL-1 activity. Deficiency in IL-1RA due to mutation results in autoinflammatory disease, underlining the critical role of IL-1RA in regulating inflammation (Aksentijevich et al., 2009). Anakinra is a recombinant, non-glycosylated form of human IL-1RA, exerts anti-inflammatory and immunomodulatory roles by binding to IL-1R and blocking its activity. The beneficial effects of anakinra have been demonstrated in the treatment of a wide range of disorders, including auto-inflammatory disorders (Cavalli and Dinarello, 2018; Kuemmerle-Deschner et al., 2020), seizure (Lai et al., 2020), pericarditis (Khayata et al., 2020), and gout (Janssen et al., 2019), wherein the pathogenesis is associated with an excessive IL-1β expression. Study has demonstrated that anakinra improved hearing loss and inflammatory in patients of atypical cryopyrin-associated periodic syndromes (Nakanishi et al., 2017). Here, we found that anakinra reduced EH, protected auditory and vestibular function, inhibited never injury, and effectively inhibited the local and systemic inflammation in a murine model of EH, which providing a new direction for clinical treatment of MD. Given that in many diseases, anakinra plays a protective role by inhibiting IL-1β signal, we speculate that anakinra alleviates EH and audiovestibular symptoms and pathological damage by inhibiting IL-1β inflammation. However, the exact and molecular mechanisms remain unexplored.

Since its introduction in 2002, thousands of patients have received anakinra for numerous indications, with a remarkable record of safety and tolerance (Cavalli and Dinarello, 2018; Janssen et al., 2019; Khayata et al., 2020; Kuemmerle-Deschner et al., 2020; Lai et al., 2020). Our study also found that anakinra inhibited weight loss induced by LPS in mice, which further validates the well-tolerated of anakinra and provides theoretical basis for future clinical trials.

Although MD is a common disease in otology, its pathogenesis is still not clear. One of the limitations of the study is that the phenotypes of EH model currently existed do not fully mimic the clinical characteristics of MD. Previous studies directly injected LPS to the scala media, although it caused obvious hearing loss, but no vestibular dysfunction (Brown et al., 2018). Intraperitoneal injection or middle ear injection of LPS resulted in inflammation in the inner ear (Bae et al., 2021; Lee et al., 2022). In this study, postauricular injection of LPS not only induced EH and serious low-to-medium frequencies hearing loss, also caused vestibular dysfunction, which comprehensively imitated the clinical symptoms of MD. Therefore, this mode of administration provided a good animal model for MD, which is beneficial to the study of the pathological mechanism, prevention and treatment of MD. However, animal studies can never replace clinical experiments, but provide basis for clinical experiments, all animal studies need be verified in more rigorous clinical trial.

As per our previous report (Zhang et al., 2022), there are elevated cytokines in the inner ear in VEO and ES of patients with MD. In addition, early study has shown that administration of LPS activated many more cytokines and provoked an inflammatory response in mice and hair cells (Fantuzzi and Dinarello, 1996; He et al., 2020). Elevated MMP3 level also affected hearing function (Nasution and Haryuna, 2019). Our study found that except IL-1β, LPS increased expression of many cytokines (*Il4*, *Il1a*, *Il6*, *Tnf*, *Ifnb*, and *Il8*) and Mmp (*Mmp3* and *Mmp13*). Interestingly, anakinra as an IL-1RA also inhibited the expression of the above genes. IL-1β upregulates the expression of many inflammatory cytokines, such as MMP3 and MMP13 (Fei et al., 2019; Singh et al., 2019). Studies show that anakinra effectively inhibited IL-8 and IL-6 secretion induced by IL-1β (Stoffels et al., 2014; Olivera et al., 2022), which is consistent with our results and suggest that anakinra plays a protective role by inhibiting these inflammatory mediators. Do these inflammatory mediators play a role in LPS-induced auditory and vestibular dysfunction? Case report has shown that Omalizumab by targeted immunoglobulin E completely resolved debilitating vertigo and nausea without any associated side effects in MD (Siebenhaar et al., 2007). Does targeted these inflammatory mediators (such as IL-6 receptor blockers sarilumab, anti-TNF agent infliximab) to regulate immune balance have a therapeutic effect on MD? The effectiveness of these immunotherapies will further verify that MD is an immune mediated disease.

In this study, we found that most of SGNs in the control group were enveloped by MBP^+^ myelin sheath. In contrast, in the LPS group, disorganization and degeneration of the myelin sheath in the cochlear nerve as well as the loss of SGNs were shown. In all complex nervous systems, neurons coexist with glial cells, which suggests that neuron-glial interaction is a main feature of neurological function. In the peripheral niveous nervous system, Schwann cells not only myelinate axons and neuron bodies, but also play an important role in maintaining the long-term functional integrity and survival of neuron (Nave and Trapp, 2008). Unlike in human, the somata of most mouse SGNs are myelinated (Xing et al., 2012). Importantly, loss of Schwann cells and intact myelin causes progressive axon degeneration and local inflammation, contributing to various peripheral neuropathies (Bourque et al., 2015). Thus, it is highly likely that the LPS-induced loss of myelin sheath integrity contributes to the degeneration of SGNs and declines in cochlear nerve function. Overload of IL-1β secretion have been shown to promote demyelination and deteriorate the severity of inflammatory neurodegenerative experimental autoimmune encephalomyelitis (Inoue et al., 2012; Huang et al., 2019). In our study, anakinra effectively alleviated cochlear nerve injury and reduced the loss of MBP by inhibiting IL-1β response, which further proves that LPS-induced neurodegeneration is at least partly mediated by IL-1β.

Cochlear nerve and vestibular nerve are very similar in terms of anatomy and development (Jeffery and Spoor, 2004). Both cochlear and vestibular nerve exhibited similar damage following exposure to ototoxic agent (Ye et al., 2019). Therefore, we only demonstrated the changes of the cochlear nerve, did not pay attention to the vestibular nerve. Whether vestibular dysfunction induced by LPS is caused by injury of vestibular myelin sheath and nerve fibers remains to be further investigated. Although no attempt was made to study the effect of hair cell in our study, study has shown that neuronal degeneration occurs independent of hair cell loss (Linthicum and Fayad, 2009). It is unclear whether LPS-induced myelin loss is a primary event which leads to secondary neurodegeneration, and vice versa. Further molecular and functional studies of the myelin protein are needed to better understand the causes of demyelination and axon degeneration.

In summary, our results demonstrated that anakinra attenuated EH, improved auditory and vestibular function, protected cochlear nerve in a murine model of EH. Furthermore, anakinra reduced the expressions of the inflammatory cytokines, macrophage infiltration and neurodegeneration in the cochlea of mice. These findings suggest that targeting IL-1 signaling may be a promising approach for treating MD.

## Data availability statement

The original contributions presented in this study are included in the article/Supplementary material, further inquiries can be directed to the corresponding author/s.

## Ethics statement

The animal study was reviewed and approved by Shandong Provincial ENT Hospital, Cheeloo College of Medicine, Shandong University Ethics Committee.

## Author contributions

HW, DZ, and NL designed the study. NZ, JL, and YLiu performed experiments. NZ and NL drafted the manuscript. SW and JG analyzed the data. XL, YS, LK, and WX housed the mice. All authors revised and approved the final version of the manuscript.
